# Incorporating healthcare access and equity in economic evaluations: a scoping review of guidelines

**DOI:** 10.1017/S0266462324000618

**Published:** 2024-11-18

**Authors:** Bryony Dawkins, Bethany Shinkins, Tim Ensor, David Jayne, David Meads

**Affiliations:** 1 Academic Unit of Health Economics, Leeds Institute of Health Sciences, University of Leeds, Leeds, UK; 2 Division of Health Sciences, Warwick Medical School, University of Warwick, Coventry, UK; 3 Nuffield Centre for International Health and Development, Leeds Institute of Health Sciences, University of Leeds, Leeds, UK; 4 Leeds Institute of Medical Research at St James’s, University of Leeds, St James’s University Hospital, Leeds, UK

**Keywords:** Equitable access to healthcare, Universal Health Coverage (UHC), Economic evaluation, Methods guidelines, Health technology assessment

## Abstract

**Background:**

International development agendas increasingly push for access to healthcare for all through universal healthcare coverage. Health economic evaluations and health technology assessment (HTA) could provide evidence to support this but do not routinely incorporate consideration of equitable access.

**Methods:**

We undertook an international scoping review of health economic evaluation and HTA guidelines to examine how well issues of healthcare access and equity are represented, evidence recommendations, and gaps in current guidance to support evidence generation in this area. Guidelines were sourced from guideline repositories and websites of international agencies and organizations providing best practice methods guidance. Articles providing methods guidance for the conduct of HTA, or health economic evaluation, were included, except where they were not available in English and a suitable translation could not be obtained.

**Results:**

The search yielded forty-seven national, four international, and nine independent guidelines, along with eighty-six articles providing specific methods guidance. The inclusion of equity and access considerations in current guidance is extremely limited. Where they do feature, detail on specific methods for providing evidence on these issues is sparse.

**Discussion:**

Economic evaluation could be a valuable tool to provide evidence for the best healthcare strategies that not only maximize health but also ensure equitable access to care for all. Such evidence would be invaluable in supporting progress towards universal healthcare coverage. Clear guidance is required to ensure evaluations provide evidence on the best strategies to support equitable access to healthcare, but such guidance rarely exists in current best practice and guidance documents.

## Key messages

- Definitions of health technology assessment often include reference to access and equity issues, yet health technology assessment guidelines rarely specify how to incorporate these impacts.
- Where access to care and equity are discussed in guidelines, they predominantly recommend that these issues should be considered separately to the main part of the HTA which should focus on the main goal of health systems: health gain.
- Clear methodological guidance is required to ensure evaluations of healthcare provide evidence on the best strategies to support equitable access to healthcare for all who need it.

## Introduction

Universal health coverage (UHC) is a global goal introduced as part of the sustainable development goals (SDGs) target 3.8, which focuses on achieving UHC, “including financial risk protection, access to quality essential health-care services and access to safe, effective, quality and affordable essential medicines and vaccines for all” [1]. UHC means all people have access to health services when and where they need them, without financial hardship, and recognizes access to healthcare as a basic human right. It is a goal that countries around the world are striving to achieve, though each has a different path to achieve it depending on the needs of the population and resources available [1;2].

Inequalities in health prevail around the world and refer to differences in health outcomes achieved by different groups in a population. Health inequities occur when these differences in health across the population are considered unfair, for example when health inequalities arise due to social conditions in which people are born, grow, live, work and age. As such health inequities are health inequalities that are unfair and could be reduced by government policies or interventions [3]. UHC continues to be challenged by health inequalities as aggregated country-level data masks within country inequalities in coverage and associated health outcomes [2].

Generally, the aim of any healthcare system is to improve the health of the population. However, with the introduction of the goal to achieve UHC, international development agendas are stretching the definition of optimal healthcare provision to include equitable access. This presents new challenges for healthcare decision makers, in how to reconcile the objective of maximizing overall population health whilst achieving equitable access to healthcare for all, and for analysts in providing evidence that can best support decision-making.

Economic evaluation is an analytical technique which involves assessing the costs and effects of healthcare interventions to identify the optimal allocation of limited healthcare resources, which maximizes population health [4]. It is a key component of HTA, and the focus of many best practice guidelines [5]. These tend to be produced by methodological experts within professional societies and contain technical guidance on methods that should be employed in each evaluation. Such documents could provide methodological guidance as to the best way to incorporate issues of equity and healthcare access into economic evaluations. However, despite the potential to, standard methods of economic evaluation do not routinely incorporate objectives beyond maximizing population health [6].

Several reviews of economic evaluation guidelines have been undertaken [7–12], with most focusing on a specific geographical area. While one took a global perspective, it was limited to national guidelines only [9]. Here, we report an international scoping review of health economic evaluation and HTA guidelines to examine how well issues of healthcare access and equity are represented [13]. In addition to national guidelines, we include guidance issued by development agencies such as the WHO and the Bill and Melinda Gates Foundation (BMGF), independent guidance issued by specific organisations such as Institute for Clinical and Economic Review (ICER) and the Panel on Cost-effectiveness [14], as well as subject-specific guidance such as the ISPOR good research practices reports [15]. We focus on what recommendations are included on how to provide evidence on equitable access to healthcare, and what gaps exist in current guidance to support evidence generation in this area.

## Methods

We undertook an international review of health economic evaluation and HTA guidelines to examine how well issues of healthcare access and equity are represented. This review of methods guidance is most closely aligned to a scoping review as it aims to examine the characteristics of current literature [13]. However, there are some differences in methods to a scoping review, particularly in relation to the way searches were conducted. Usually, a scoping review would include systematic database searches (similar to a systematic review), however this search method is not applied here as most guidelines are not deposited in databases. Consequently, this review was not registered. Despite these differences, reporting of this review was guided by the PRISMA-ScR guidance and the completed PRISMA-ScR checklist is available in Supplementary Appendix 1 [16].

### Identification of guidance documents

Guidelines were sourced by searching the Guide to Economic Analysis and Research for Health (GEAR4Health) and ‘ISPOR Pharmacoeconomic Guidelines around the World’ repositories, along with websites of the WHO, BMGF, the International Decision Support Initiative (iDSI), ICER, and the Panel on Cost-effectiveness [17–19] [20–24] [25;26]. Articles providing guidance on specific methods relevant to economic evaluation and HTA were also sought by searching within the European Network for HTA (EUNetHTA) and ISPOR ‘Good Practice Reports’ series [27;28].

Initial searches were completed in January 2021 and were updated in March 2023 to include new and updated guidance.

### Eligibility criteria

Any document which identified itself as providing methodological guidance for the conduct of HTA or economic evaluation of healthcare was included. Documents not available in English were included if an acceptable translation could be obtained using Google Translate. Full inclusion and exclusion criteria are presented in Table 1.

**Table 1.** Inclusion and exclusion criteria

Include	Exclude
*Type of document*	
National or international guidelines or methods guidance for health economic evaluation or HTA	Any document which does not provide methods guidance for the conduct of health economic evaluation or HTA
	Guidance documents that are relevant for a sub-national region only
*Subject of document*	
Guidance for the conduct of health economic evaluation or HTA	Discussion of health economic evaluation or HTA, without providing guidance
*Document characteristics*	
Most recent guideline available	Duplicate or outdated versions of guidelines where an updated version is available
Documents available in English or where a suitable translation can be obtained	Documents not available in English and where a suitable translation could not be obtained
*Time frame*	
Up to March 2023	None

### Data extraction and synthesis

Data were extracted using a template which collected: (i) general guideline characteristics; (ii) whether healthcare access (or related terms e.g., “use of services” or “coverage”) was mentioned and, if so, details of the discussion; (iii) whether inequalities or equity (or related terms e.g., “distribution” or “fairness”) were mentioned and, if so, details of the discussion; and, (iv) whether any relevant methods for the consideration of healthcare access or equity issues were mentioned and, if so, details of these.

Descriptive analysis of the extent to which healthcare access and equity issues are covered in the guidelines was undertaken. This sought to draw out similarities, differences, and trends across guidelines, and to identify those that made more explicit recommendations than others and what those recommendations were. Results were synthesized separately for documents providing overall guidance on the whole HTA/economic evaluation process and those providing guidance on a specific method that could be used as part of an economic evaluation.

## Results

A total of fifty national, eight international, twenty-six independent, and eighty-six method-specific guideline documents were identified by the search (see Supplementary Appendix 2). Three national guidelines were excluded. One of these (Iran) was excluded as it was not available online and could not be sourced by alternative means. Another (Catalonia) was excluded as it was determined to be regional, and the national guideline for Spain was included. One more (South Korea) was excluded as the translation was not of sufficient quality to interpret the data. A further twenty-one articles were excluded at full text review as they did not provide methods guidance.

**Figure 1.**
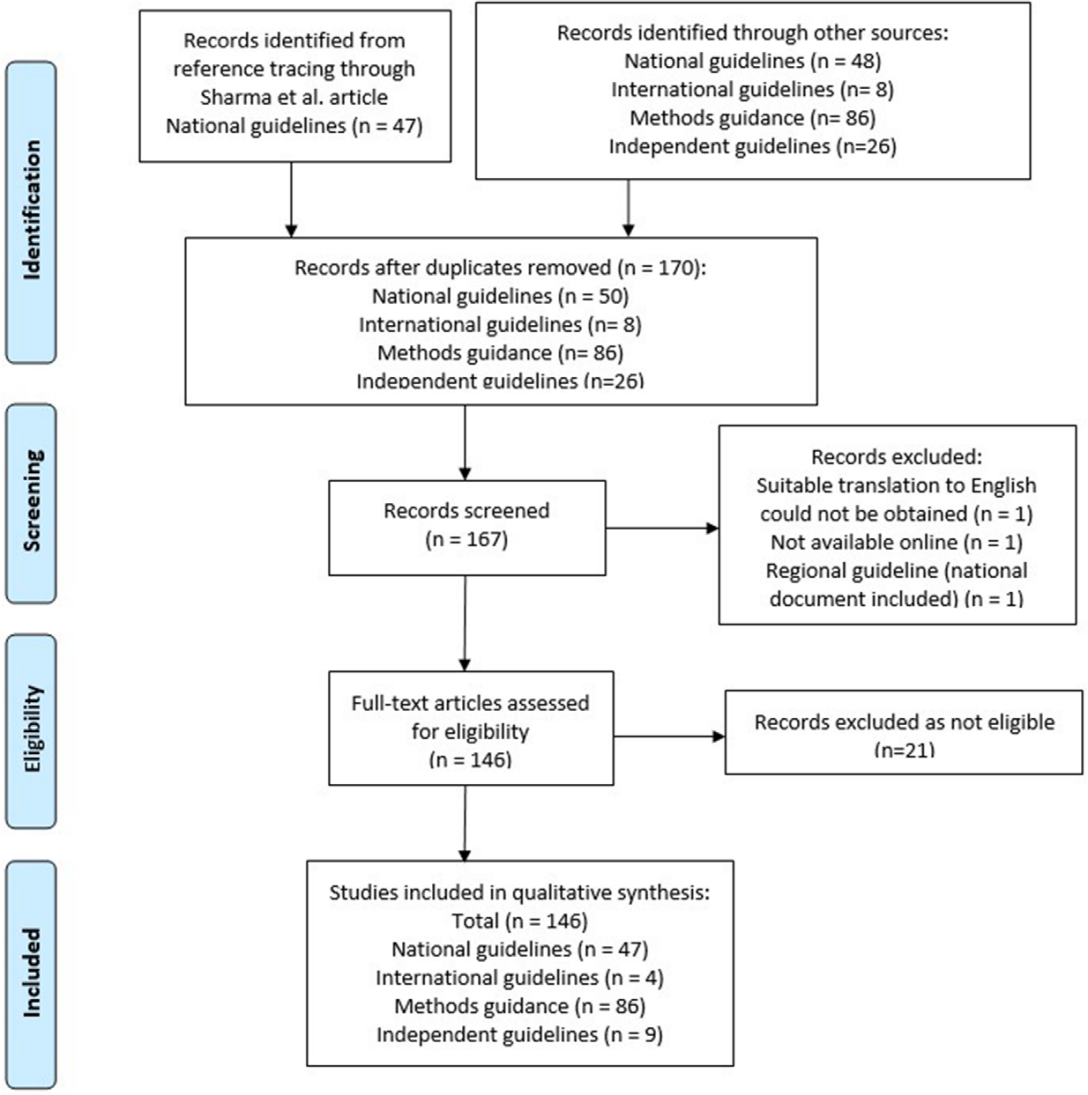
Prisma flow diagram.

A total of 146 guideline documents were included in this review. The PRISMA flow diagram in Figure 1 shows the selection process.

### General characteristics

**Figure 2.**
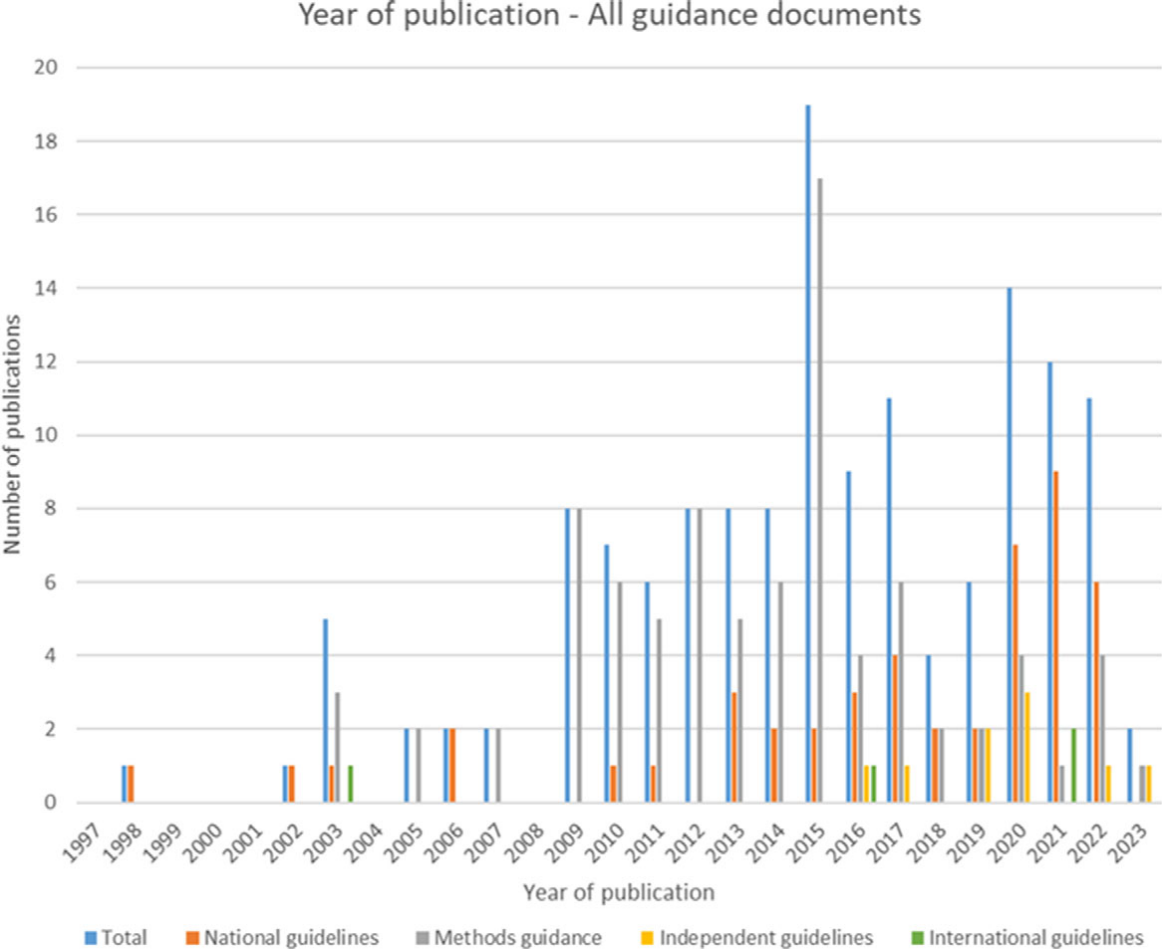
Year of publication – all guidance documents.

**Figure 3.**
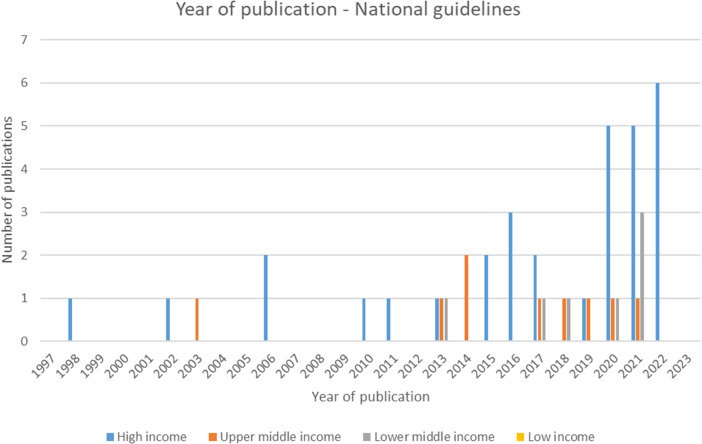
Year of publication – national guidelines.

Of the forty-seven included national guidelines, thrity-one provided guidance for high-income countries (HICs), nine for upper-middle-income countries (U-MICs), and seven for lower-middle-income countries (L-MICs), according to World Bank income classification groupings. The distribution of guidance document types by publication year is shown in Figure 2 and the distribution of national guidelines published by year and income classification is shown in Figure 3. Most guidance documents were published since 2010 and for HICs. Guidance produced for middle-income countries (MICs) is increasing, but there were no national guidelines for low-income countries (LICs).

### Healthcare access

#### Summary of healthcare access in current guidance

**Figure 4.**
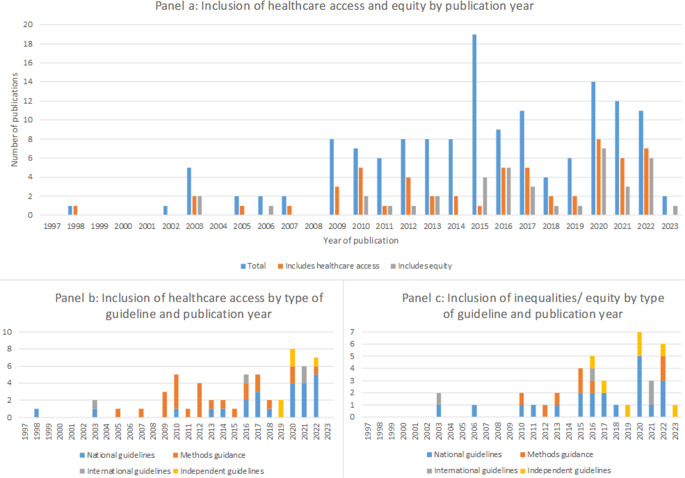
Inclusion of healthcare access and equity in current guidance.

Healthcare access was mentioned in 40 percent of all guidance documents (Figure 4). Consideration of healthcare access has increased in recent years and is most commonly included in guidance for lower resource settings. Overall, forty-five percent (*n* = 14/31) of all guidelines from HICs, fifty-six percent (*n* = 5/9) from U-MICs and seventy-one percent (*n* = 5/7) from L-MICs included some discussion of healthcare access.

#### Healthcare access in documents providing overall guidance for economic evaluation and HTA

All international guidelines, fifty-one percent of national guidelines and fifty-five percent of independent guidelines mention healthcare access. However, generally the discussion of healthcare access was extremely limited, with little practical detail provided [29;30]. In some cases healthcare access was not discussed explicitly but access-related indicators such as treatment rates, implementation, uptake, and compliance were requested [30–32], or access schemes were discussed [33]. In others, discussion of healthcare access was limited to recommending that access information should be included in the description of the product/disease, introductory or discussion sections [34–37], or as one (of potentially many) socio-cultural aspects that should be considered alongside clinical and economic evidence [35;37–41]. In many cases, healthcare access is discussed as an optional consideration and so is not included within the decision-making process [37;38;41]. Furthermore, even when guidelines advocate considering healthcare access, they rarely provide guidance on what evidence should be provided, how it should be obtained, or how it should be incorporated into the decision-making process [35;36;42–45].

Within guidance documents from independent organizations, discussion of healthcare access is limited. It is not mentioned at all in the 2nd Panel on cost-effectiveness article and while five of the eight included ICER documents include some discussion of healthcare access, beyond recognizing access as a social objective, this is predominantly related to how access can be restricted if high prices would mean unmanageable budget impacts [14;46–50].

Three international guidelines and three national guidelines, all from LMICs, specifically explain the importance of the role of HTA in achieving UHC [51–56]. However, most give little detail on the way this guidance should be delivered, how evaluations should incorporate issues or how evidence to support these goals should be (i) generated and (ii) balanced against other healthcare system objectives [51–54]. For example, the iDSI reference case advocates consideration of equity and for robust economic evaluation to aid with decision making to support achievement of UHC. However, it makes no methodological suggestions about providing evidence to support UHC other than through robust economic evaluation “conducted and reported with consistently high methodological quality” [54]. In contrast, the guidelines from the WHO CHOICE series do recommend some evaluative approaches to support UHC, including the use of generalized cost-effectiveness analysis for developing UHC packages [55;56]. The guideline for the Philippines includes the most comprehensive discussion of healthcare access among the included national and international guidelines, providing practical recommendations on how evidence should be collected and presented, and how this evidence should be used within the decision-making process [52]. Besides explaining the importance of HTA in the achievement of UHC, it also mandates the consideration of evidence on equity and access and recommends a range of qualitative methods to collect data for this purpose [52]. Still, healthcare access is considered separately to clinical and economic data.

#### Healthcare access in methods guidance articles

One article from EUNetHTA and 24 ISPOR Good Practice Reports contained discussion of healthcare access [57–81]. However, most simply describe healthcare access as a possible issue rather than suggesting methods to analyze it. For example, treatment adherence is discussed as potentially impacting effectiveness and costs [57], in some articles factors which potentially limit access are described [64;67], and in some the impact of policies or provider types on access are described [61;69;70;82]. Others suggest factors impacting access to the intervention should be considered, but do not say how [59;66;78;81]. However, some practical suggestions for incorporating access into analyses are suggested. For example, a societal perspective for analysis is suggested to facilitate coverage decisions to provide access to more patients [71]; stratification and weighting methods are recommended to overcome selection bias resulting from differences in utilization by different populations [81]; dynamic simulation, discrete event simulation, machine learning models and constrained resource modelling/constrained optimization are suggested to capture changes/constraints in healthcare access [58;74;81;83;84]; regression-based decomposition methods are suggested for examining disparities in access to healthcare [65]; and multiple-criteria decision analysis is suggested to incorporate improvements in access within the evaluation [75]. Further explanation of the methods identified from the review that could be useful for the analysis of healthcare access is provided in Supplementary Appendix 3.

### Inequalities and equity

#### Summary of inequalities/equity in current guidance

Inequalities and equity issues are mentioned in twenty-seven percent of documents identified (Figure 4). This shows that, like healthcare access, the inclusion of some discussion on inequalities and equity has been increasing in recent years. However, in contrast to healthcare access this is primarily in national and international guidelines with only a small proportion being specific methods guidance.

#### Inequality and equity in documents providing overall guidance for economic evaluation and HTA

Discussion of equity issues in many guidance documents remains limited to short statements of the importance of equity without making any attempt to explain this or how it should be included in analyses [31;42;45;53;55;56;85–91].

The most common recommendation is to ensure all QALYs are weighted equally regardless of to whom they accrue [31;40;42;86;89;91–94], although some allow alternative equity positions to be presented if useful for decision making [31;40;43]. The guideline for Canada also makes recommendations of how to do this, including presenting disaggregated analyses along with “full descriptions of the relevant populations to allow for consideration of any subsequent distributional and equity-related policy concerns” [43].

Another common recommendation is for distributional inequities to be discussed alongside, but separately, to the main clinical and economic analysis, for example, using subgroup analysis or equity/ distributional weights [39;41;42;54;93]. For example, the Scottish guideline states that equity implications should be discussed, noting that although “[i]t can be difficult to include equity considerations within an economic evaluation”… “[t]hey can certainly be included in a discussion of the main findings” [33]. Other guidelines take a similar approach, suggesting a descriptive approach to inclusion of equity considerations and distributional impacts [36;37;86;89]. One justification being that equity is considered “less quantifiable” despite influencing decision making [37]. Some guidelines discuss equity weighting, though the majority advise against use of this method due to methodological issues in the derivation of equity weights and unresolved debate about the appropriateness of this technique [86;89].

Similar to the inclusion of healthcare access, the guideline for the Philippines also stands out for its explicit inclusion of equity issues. It states that equity considerations and social values are central principles for the conduct of HTA [52]. It also explains that ethical issues including “equity and fairness of coverage decisions; considerations for special subgroups of patients and the general population, where applicable” and “social acceptability and cultural factors including patient and caregiver preferences and values” should be included as some of the “key aspects of evidence which must form the HTA report” [52]. Furthermore, it recommends methods for incorporating these issues, suggesting a separate section on equity organized according to PROGESS-Plus (a framework for specifying indicators of social disadvantage) [52;95].

All international guidelines emphasize the importance of equity as a goal of the healthcare system to be considered alongside efficiency/cost-effectiveness [30;54–56]. In addition, the iDSI reference case suggests that equity implications should be considered at all stages of the evaluation and that there are various mechanisms for assessing equity implications, though it does not state what these are [54].

Within guidance documents from independent organizations the discussion of equity is mostly limited to acknowledging equity is one of many factors to be considered alongside cost-effectiveness in resource allocation decisions [14;47;49;50;96;97]. The exception to this is the ICER White Paper on “Advancing health technology assessment methods to support health equity”, which comprehensively addresses the inclusion of equity within HTA, advocating for inclusion of equity through deliberative process [98]. It recommends standardization of evidence on equity to be presented to decision makers as part of the HTA process and promotes the use of empirical evidence on equity to support, but not replace, the deliberative process. It also discusses a range of methodological options to provide evidence on equity to inform the deliberative process and makes recommendations about the use of each [98]. As such, this article represents a marked shift forward in the way equity is reflected in guidance for economic evaluation and HTA.

#### Inequality and equity in methods guidance articles

Two articles from the EUNetHTA series and six from the ISPOR Good Practice Reports series contained discussion of inequalities or equity [60;75;77;82;99–102]. The discussion is limited to describing equity implications as being potentially relevant for consideration [77;99;101;102]. Within the ISPOR Good Practice Reports, equity issues also arise with respect to equity of competition [72], equity adjustments which could influence characterization of health [60], and as one of five criteria considered within an example multi-criteria decision analysis from Thailand [75]. Although there is limited discussion of equity issues within methods guidance, several recommend subgroup analysis or disaggregation of data when there may be differences in results for different population groups [66;78;103–106]. Overall, recognition of equity issues is limited in methods guidance articles, and the only concrete example of equity issues being formally incorporated into analysis is through multi-criteria decision analysis. One exception to this is the ISPOR CHEERS checklist which was updated in 2022 and now specifically includes criteria relating to equity and distributional analysis [100]. It recommends that characterizing distributional effects may be important and suggests that methods for assessing trade-offs between equity and efficiency should be described, for example, using the equity-efficiency impact plane [100]. Further explanation of the methods identified from the review that could be useful for the analysis of equity impacts is provided in Supplementary Appendix 3.

## Discussion

### Summary of key findings and implications

This review was undertaken to examine how well issues of healthcare access and equity are represented in current guidance, what recommendations are included to provide evidence on these issues, and what gaps exist. It provides the first comprehensive account of how well current economic evaluation and HTA methods guidance supports evidence generation for healthcare policy and practice that would result in progress towards UHC and fair access to healthcare.

The results show that despite these being prioritized issues in international development agendas, the majority of guidelines do not mention healthcare access or equity at all. As such, they fail to recognize patients’ ability to access care as a key outcome that should be measured or evaluated. Even those that do advocate for the importance of healthcare access fail to provide guidance on specific methods. Equity issues are mentioned in only 27 percent of included guidance documents. Where they are, they primarily advocate for maximization of unweighted QALYs or suggest further consideration should be separate to the main analysis. Despite increasing recognition of the need to consider equity issues, there remains a lack of practical guidance on how they should be reported and incorporated into healthcare decision-making. One exception to this is the ICER White Paper which represents a major shift in the equity stance of ICER as an organization. It places equity as an equal consideration to cost-effectiveness in healthcare decision making and introduces a range of methods and minimum standards by which to do this. Given the system requirements needed to deliver equitable access to healthcare, it will be interesting to see the extent to which the new ICER guidance translates to improved equity of access in practice.

A summary of all methods identified from the review that could be useful for the analysis of healthcare access and equity within economic evaluation and HTA is provided in Supplementary Appendix 3. Some of the methods identified are discussed in current guidance in relation to their usefulness for analysis of either equity or access issues but rarely is a method discussed in relation to both these aspects. For example, the ICER White Paper discusses a number of methodological options for analyzing equity but does not discuss healthcare access considerations. In contrast, the WHO CHOICE articles discuss methods in relation to identifying strategies for universal health coverage but do not specifically discuss methods for incorporating equity. As such, analysts who need to analyze equity and access impacts together likely need to select appropriate methods for each and consider how they might combine them to provide comprehensive evidence on both aspects. An additional challenge to be overcome is that the more comprehensive methods to incorporate equity or access issues require increasingly comprehensive data which in many settings simply does not exist and can be impractical to obtain. In response to this issue there have been some attempts to develop less resource intensive methods for analysis of equity such as aggregate distributional cost-effectiveness analysis and the health improvement distribution index (see Supplementary Appendix 3); however, even these require additional data resource compared with standard methods used within economic evaluation and HTA.

The number of national guideline documents for U-MICs and L-MICs have increased over time, however, still the largest proportion of national guidelines are for HICs, and to date there are no national guidelines for low-income countries. These trends are understandable as HICs have tended to spearhead the use of health economic evidence for health decision making and have more robust systems for incorporating this evidence in the decision-making process. In addition, weaker health systems in LMICs can hinder the use of evidence from economic evaluation and HTA to inform healthcare decision making due to the need to overcome complex challenges throughout care pathways (e.g., in purchasing and supply chains, and ensuring appropriate targeting and provision to patients). However, there has also been a shift in international development focus to move toward increased use of health economic evidence and HTA processes in LMICs which reflects the increase in guidance documents more recently [6]. Furthermore, included guidelines for some of the L-MICs contain some of the most detailed guidance on healthcare access and equity issues. This potentially reflects the greater emphasis for these countries on achieving UHC as compared with HICs which have more comprehensive healthcare systems and generally can provide basic healthcare services for their populations.

The results of this study highlight a disparity between international development agenda expectations for use of health economic evidence to support advancement towards UHC and the current methods guidance to undertake such evaluations. The World Health Assembly report on ‘health intervention and technology assessment in support of universal health coverage’ advocates for robust economic evaluation and HTA to support advances towards UHC, yet does not provide guidance on how economic evaluation should be adapted to provide relevant evidence [6]. Furthermore, the statement that “robust economic evaluation is critical for UHC” is repeated through several guidance documents. Yet, in all cases, no additional guidance on the role of economic evaluation in supporting UHC is given. This implies that robust economic evaluation by itself will support UHC. To some extent this could be true if increased overall health means increased access to care. However, increased health could also be achieved by improving treatments for people already able to access care. As such, even when conducted robustly, economic evaluation does not necessarily provide evidence on advances towards UHC. Such evidence would require adaptations to standard economic evaluation methods to incorporate these issues. This problem is not addressed in current national and international guidelines for economic evaluation and HTA and, as such, no guidance on how to effectively provide relevant evidence on advancement towards UHC is provided in current guidelines.

### Strengths and limitations

This study is the first to examine recommendations within current methods guidance which would support the generation of evidence on achieving UHC and fair access to healthcare for all. As such, it provides novel evidence on how well-aligned current methods guidance is with international development agendas, and how well current recommended methods can provide the evidence needed to support advancement towards global goals.

Another strength of this study is that it was conducted robustly using methods to identify guideline documents in line with other published studies in this area. In addition, every effort was made to include articles produced in languages other than English. In contrast, previous reviews of methods guidance have included only articles available in English resulting in a large proportion of known guidance documents being excluded [9].

A limitation of this study (and of guideline reviews in general) is that because economic evaluation and HTA guidelines tend not to be published in journal articles the search methods are non-standard as compared to other types of literature review. Instead of using a structured literature search within relevant databases, guidelines are identified from online repositories and by searching websites of relevant organizations. Although this approach is common among guideline reviews it is potentially less re-producible and transparent. Furthermore, quality assessment of the included guidance documents was not undertaken because of the variation in type of document included, and also because there is no validated tool available to assess the quality of economic evaluation and HTA guidelines. In any case, the quality of the guidelines was not the intended purpose of this review.

## Conclusion

Clear methodological guidance is required to ensure evaluations of healthcare provide evidence on the best strategies to support equitable access to healthcare, but such guidance rarely exists in current best practice and guidance documents. Current recommendations are vague and fail to provide specific methodological guidance on how these important issues can be incorporated into economic evaluations. As such, current guidance fails to support evidence generation, from economic evaluations and HTA, which could be used by policy makers around the world as they seek to progress towards UHC. We hope that highlighting this gap will encourage discussion of how methods of economic evaluation and HTA could be adapted to provide this evidence.

## Supporting information

Dawkins et al. supplementary material 1 Dawkins et al. supplementary material

Dawkins et al. supplementary material 2 Dawkins et al. supplementary material

Dawkins et al. supplementary material 3 Dawkins et al. supplementary material

## Supplementary material

The supplementary material for this article can be found at http://doi.org/10.1017/S0266462324000618.
