# Supplementary material for: Incorporating healthcare access and equity in economic evaluations: a scoping review of guidelines
Source: Int J Technol Assess Health Care. 2024 Nov 18;40(1):e59. doi: 10.1017/S0266462324000618 (PMC11579673; doi:10.1017/S0266462324000618)
Supplement: Dawkins et al. supplementary material 2 — Dawkins et al. supplementary material [file S0266462324000618sup002.docx]

# Supplementary Appendix 2 – Included and excluded articles

## Included articles

### National guideline documents = 47

1. Pharmaceutical Benefits Advisory Committee (PBAC), *Guidelines for preparing a submission to the Pharmaceutical Benefits Advisory Committee (Version 5.0)*, ed. Australian Government - Department of Health. 2016, Australia: Australian Government: Department of Health.
2. Evelyn Walter and Susanne Zehetmayr, *Guidelines on Health Economic Evaluation: Consensus paper*. 2006, Institute for Pharmacoeconomic Research: Vienna, Austria.
3. Daiga Behmane, et al., *Baltic Guideline for Economic Evaluation of Pharmaceuticals (Pharmacoeconomic Analysis)*. 2002, Baltic.
4. Irina Cleemput, et al., *Belgian Guidelines for Economic Evaluations and Budget Impact Analyses: Second Edition*. 2015, Brussels: Belgian Healthcare Knowledge Centre (KCE).
5. Essential Medicines and Technology Division (EMTD): Department of Medical Services: Ministry of Health, *Health Technology Assessment Process Guideline (2nd edition)*. 2018, Bhutan: Ministry of Health.
6. Secretariat of Science Technology and Strategic Inputs: Department of Science and Technology, *Methodological Guidelines: Economic Evaluation Guideline (2nd Edition)*. 2014, Brazil: Ministry of Health.
7. CADTH Methods and guidelines, *Guidelines for the Economic Evaluation of Health Technologies: Canada (4th Edition)*. 2017, Ottawa: CADTH.
8. *China Guidelines for Pharmacoeconomic Evaluations 2020 Edition*, G.G. Liu, Hu, S., Wu, J., Wu, J., Dong, Z., Li, H., Editor. 2020, Sponsored by: Chinese Pharmaceutical Association, China Society for Pharmacoeconomics and Outcomes Research: China.
9. Moreno Viscaya, M., Mejía Mejía, Aurelio, Castro Jaramillo, Héctor Eduardo, *Manual for the elaboration of economic evaluations in health (translated title)*. 2014, Colombia: Institute of Technological Evaluation in Health (IETS).
10. Agency for Quality and Accreditation in Health Care: Croatia: Department for Development Research and Health Technology Assessment, *The Croatian Guideline for Health Technology Assessment Process and Reporting, 1st edition*. 2011, Zagreb, Croatia: Agency for Quality Accreditation in Health Care.
11. González, A.M.G., *Methodological guide for Economic Evaluation in Health. Cuba.* 2003, Havana: Ministry of Public Health - National School of Public Health.
12. Klimes, J., Mlÿoch, T., Pásztor, B., Baloghová, K., Veselá, S., Bulejová. L., Tužil, J., Ornstová, E., Chadimová, K., Decker, B., Bartáková, J, Doležal, T.,, *Recommended procedures for health economic evaluations in the Czech Republic: Czech Society for Pharmacoeconomics and Health technology evaluation [Translated title]*. 2020: Czech Republic.
13. Danish Medicines Council, *The Danish Medicines Council methods guide for assessing new pharmaceuticals*. 2021: Denmark.
14. Pharmacoeconomic Unit: Central Administration for Pharmaceutical Affairs, *Guidelines for Reporting Pharmacoeconomic Evaluations (Version: 01)*. 2013, Egypt: Egyptian Drug Authority, Ministry of Health.
15. National Institute of Health and Care Excellence [NICE], *NICE health technology evaluations: the manual*. 2022, London: NICE.
16. Pharmaceuticals Pricing Board Finland, *Application instructions: Application for basic/ special reimbursement status and reasonable wholesale price for a medicinal product subject to marketing authorization*. 2021: Finland.
17. Haute Autorité de Santé, *Methodological Guidance: Choices in Methods for Economic Evaluation*. 2020: France.
18. Institute for Quality and Efficiency in Healthcare (IQWiG), *General Methods: Draft version 7.0 [translated title]*. 2022: Germany.
19. Ministry of Human Resources, *Health professional guideline - For preparing and evaluating health economic analyses [translated title]*. 2021: Hungary.
20. Department of Health Research Ministry of Health & Family Welfare Government of India, *Health Technology Assessment in India (HTAIn) And DHR-ICMR Advanced Molecular Oncology Diagnostic Services (DIAMOnDS) Guidelines*. 2021: India.
21. Indonesian Health Technology Assessment Committee (InaHTAC), *Health Technology Assessment (HTA) Guideline*. 2017, Jakarta: Ministry of Health, Indonesia.
22. Health Information and Quality Authority, *Guidelines for the Economic Evaluation of Health Technologies in Ireland*. 2020: Ireland.
23. National List of Health Services (NLHS) Assessment Division, *Procedure for submitting an application for the inclusion of a preparation ("medicine") in the basket of health services (according to State Health Insurance Law, 1994) [translated title]*. 2022, Ministry of Health: Israel.
24. Agenzia Italiana Del Farmaco, *Guidelines for the compilation of the dossier in support of the request of refundability and price of a medicinal product [translated title].* 2020.
25. Centre for Outcomes Research and Economic Evaluation for Health (CORE2 HEALTH), *Guideline for preparing cost-effectiveness evaluation to the central social insurance medical council*. 2022, National Institute of Public Health: Japan.
26. Pharmacy Practice and Development Division, *Pharmacoeconomic Guidelines for Malaysia*. 2019, Malaysia: Ministry of Health Malaysia.
27. Inter-institutional Commission of the Basic Table of Inputs of the Health Sector, *Guide for conducting economic evaluation studies to update the basics table and catalog of supplies of the health sector in Mexico [translated title]*. 2017, General Health Council: Mexico.
28. Pharmaceutical Management Agency (New Zealand) (PHARMAC), *Prescription for Pharmacoeconomic Analysis: Methods for cost-utility analysis (Version 2.2)*. 2015, New Zealand: PHARMAC.
29. The Norwegian Medicines Agency (NoMA), *Guidelines for the submission of documentation for single technology assessment (STA) of pharmaceuticals*. 2021: Norway.
30. Health Technology Assessment Unit: Department of Health, *Philippine HTA Methods Guide, First Edition*. 2020, Republic of the Philippines: Department of Health.
31. The Agency for Health Technology Assessment and Tariff System, *Health Technology Assessment Guidelines (version 3.0)*. 2016, Warsaw: The Agency for Health Technology Assessment and Tariff System.
32. Da Silva, E.A., et al., *Guidelines for economic drug evaluation studies*. 1998, Lisbon: National Authority of Medicines and Health Products [INFARMED].
33. Omelyanovsky, V.V., Avksentieva, M.V., Sura, M.V., Khachatryan, G.R., Gerasimov, K.V., Ivakhnenko, O.I., Dzanaeva, A.V., *Methodological recommendations for conducting a comparative clinical and economic assessment medicine [translated title]*. 2018, Federal State Budetary Institution, Ministry of Health of the Russian Federation: Moscow.
34. Scottish Medicines Consortium (SMC), *Guidance to submitting companies for completion of New Product Assessment Form (NPAF)*. 2022, Healthcare Improvement Scotland: Scotland.
35. *Drug evaluation methods and process guide*. 2019, Singapore: Agency for Care Effectiveness (ACE).
36. The Ministry of Health of the Slovak Republic, *Declaration 422 of the Ministry of Health of the Slovak Republic on the details of the pharmaco-economic analysis of the drug: Temporary version of the regulation effective from June 1, 2021 [translated title]*. 2021: Slovak Republic.
37. *Rules on the classification of medicinal products [Translated title]*. 2013, Slovenia: Official Gazette of the RS.
38. Department of Health, *Guidelines for Pharmacoeconomic Submissions*. 2012, Republic of South Africa: Department of Health.
39. López-Bastida, J., et al., *Spanish recommendations on economic evaluation of health technologies.* 2010. **11**(5): p. 513-520.
40. *The Swedish Dental and Pharmaceutical Benefits Agency common advice*. 2017, Sweden: The Swedish Dental and Pharmaceutical Benefits Agency.
41. *Operationalization of the criteria "effectiveness, expediency and efficiency" according to Article 32 of the Federal Law on Health insurance (KVG) [translated title]*. 2022, Federal Office of Public Health: Switzerland.
42. Taiwan Society for Pharmacoeconomic Outcomes Research (TaSPOR), *Guidelines of Methodological Standards for Pharmacoeconomic Evaluations in Taiwan (Version 1.0)*. 2006, Taiwan: TaSPOR.
43. Guideline Development Working Group, *Guideline for Health Technology Assessment in Thailand Updated Edition: 2019*, S. Pannarunothai, Pilasant, S., Kingkaew, P., Saengsri, W., Editor. 2021, Health Systems Research Institute (HSRI): Thailand.
44. *Guideline for economic evaluations in healthcare*. 2016, The Netherlands: Zorginstituut Nederland.
45. National Authority for Evaluation and Accreditation in Health (INEAS), *Assessment of health technologies. Methodological choices for pharmaco-economic studies at INEAS [translated title]*. 2021, INEAS: Tunis.
46. Ministry of Health of Ukraine, *State assessment of medical technologies for medicinal products [translated title]*. 2021: Ukraine.
47. Academy of Managed Care Pharmacy (AMCP) Format Executive Committee, *AMCP Format for Formulary Submissions: Guidance on Submission of Pre-approval and Post-approval Clinical and Economic Information and Evidence Version 4.1*. 2020, AMCP: USA.

### International guideline documents = 4

1. Bertram, M.Y., et al., *Methods for the economic evaluation of health care interventions for priority setting in the health system: an update from WHO CHOICE.* 2021. **10**(11): p. 673.
2. Bertram, M.Y., et al., *Progressive Realisation of Universal Health Coverage in Low-and Middle-Income Countries: Beyond the" Best Buys".* 2021. **10**(11): p. 697.
3. Edejer, T.T.-T., et al., *Making choices in health: WHO guide to cost-effectiveness analysis*. Vol. 1. 2003: World Health Organization.
4. Wilkinson, T., et al., *The international decision support initiative reference case for economic evaluation: an aid to thought.* 2016. **19**(8): p. 921-928.

### Independent guideline documents = 9

1. Agboola, F., M. Whittington, and S. Pearson, *Advancing Health Technology Assessment Methods that Support Health Equity*, Institute for Clinical and Economic Review (ICER), Editor. 2023.
2. Institute for Clinical and Economic Review (ICER), *Adapted Value Assessment Methods for High-Inpact "Single and Short-Term Therapies" (SSTs)*. 2019.
3. Institute for Clinical and Economic Review (ICER), *A Guide to ICER's Methods for Health Technology Assessment*. 2020.
4. Institute for Clinical and Economic Review (ICER), *ICER's Reference Case for Economic Evaluations: Principles and Rationale*. 2020.
5. Institute for Clinical and Economic Review (ICER), *Adaptations to the ICER methods for evaluation of therapies for COVID-19*. 2020.
6. Institute for Clinical and Economic Review (ICER), *Modifications to the ICER value assessment framework for treatments for ultra-rare diseases: Final version, November 2017 (Updated January 31, 2020)*. 2020.
7. Institute for Clinical and Economic Review (ICER), *2020-2023 Value Assessment Framework: January 31, 2020 (Updated February 3, 2022).* 2022.
8. Pearson, S.D., *Why the coming debate over the QALY and disability will be different.* 2019. **47**(2): p. 304-307.
9. Sanders, G.D., et al., *Recommendations for conduct, methodological practices, and reporting of cost-effectiveness analyses: second panel on cost-effectiveness in health and medicine.* 2016. **316**(10): p. 1093-1103.

### Method-specific guidance documents = 86

1. Motheral, B., et al., *A checklist for retrospective database studies—report of the ISPOR Task Force on Retrospective Databases.* 2003. **6**(2): p. 90-97.
2. Berger, M.L., et al., *A questionnaire to assess the relevance and credibility of observational studies to inform health care decision making: an ISPOR-AMCP-NPC Good Practice Task Force report.* 2014. **17**(2): p. 143-156.
3. Crown, W., et al., *Application of constrained optimization methods in health services research: report 2 of the ISPOR optimization methods emerging good practices task force.* 2018. **21**(9): p. 1019-1028.
4. Marshall, D.A., et al., *Applying dynamic simulation modeling methods in health care delivery research—the SIMULATE checklist: report of the ISPOR simulation modeling emerging good practices task force.* 2015. **18**(1): p. 5-16.
5. Sullivan, S.D., et al., *Budget impact analysis—principles of good practice: report of the ISPOR 2012 Budget Impact Analysis Good Practice II Task Force.* 2014. **17**(1): p. 5-14.
6. Walton, M.K., et al., *Clinical outcome assessments: conceptual foundation—report of the ISPOR clinical outcomes assessment–emerging good practices for outcomes research task force.* 2015. **18**(6): p. 741-752.
7. Powers III, J.H., et al., *Clinician-reported outcome assessments of treatment benefit: report of the ISPOR clinical outcome assessment emerging good practices task force.* 2017. **20**(1): p. 2-14.
8. European Network for Health Technology Assessment, *Comparators & Comparisons. Criteria for the choice of the most appropriate comparator (s). Summary of current policies and best practice recommendations. Final version [Internet]. Copenhagen: EUnetHTA, 2013.* 2015 (Adapted version).
9. European Network for Health Technology Assessment, *Comparators & Comparisons. Direct and indiriect comparisons. Final version [Internet]. Copenhagen: EUnetHTA, 2013.* 2015 (Adapted version).
10. Roberts, M., et al., *Conceptualizing a model: a report of the ISPOR-SMDM modeling good research practices task force–2.* 2012. **32**(5): p. 678-689.
11. Hoaglin, D.C., et al., *Conducting indirect-treatment-comparison and network-meta-analysis studies: report of the ISPOR Task Force on Indirect Treatment Comparisons Good Research Practices: part 2.* 2011. **14**(4): p. 429-437.
12. Bridges, J.F., et al., *Conjoint analysis applications in health—a checklist: a report of the ISPOR Good Research Practices for Conjoint Analysis Task Force.* 2011. **14**(4): p. 403-413.
13. Husereau, D., et al., *Consolidated health economic evaluation reporting standards (CHEERS)—explanation and elaboration: a report of the ISPOR health economic evaluation publication guidelines good reporting practices task force.* 2013. **16**(2): p. 231-250.
14. Crown, W., et al., *Constrained optimization methods in health services research—an introduction: report 1 of the ISPOR optimization methods emerging good practices task force.* 2017. **20**(3): p. 310-319.
15. Johnson, F.R., et al., *Constructing experimental designs for discrete-choice experiments: report of the ISPOR conjoint analysis experimental design good research practices task force.* 2013. **16**(1): p. 3-13.
16. Patrick, D.L., et al., *Content validity—establishing and reporting the evidence in newly developed patient-reported outcomes (PRO) instruments for medical product evaluation: ISPOR PRO good research practices task force report: part 1—eliciting concepts for a new PRO instrument.* 2011. **14**(8): p. 967-977.
17. Patrick, D.L., et al., *Content validity—establishing and reporting the evidence in newly developed patient-reported outcomes (PRO) instruments for medical product evaluation: ISPOR PRO Good Research Practices Task Force report: part 2—assessing respondent understanding.* 2011. **14**(8): p. 978-988.
18. Ramsey, S.D., et al., *Cost-effectiveness analysis alongside clinical trials II—an ISPOR Good Research Practices Task Force report.* 2015. **18**(2): p. 161-172.
19. Pitman, R., et al., *Dynamic transmission modeling: a report of the ISPOR-SMDM modeling good research practices task force-5.* 2012. **15**(6): p. 828-834.Mauskopf, J., et al., *Economic analysis of vaccination programs: an ISPOR good practices for outcomes research task force report.* 2018. **21**(10): p. 1133-1149.
20. European Network for Health Technology Assessment, *Endpoints used for Relative Effectiveness Assessment: Clinical Endpoints. Final version [Internet]. Copenhagen: EUnetHTA, 2013.* 2015 (Adapted version).
21. European Network for Health Technology Assessment, *Endpoints used for Relative Effectiveness Assessment: Composite endpoints. Final version [Internet]. Copenhagen: EUnetHTA, 2013.* 2015 (Adapted version).
22. European Network for Health Technology Assessment, *Endpoints used for Relative Effectiveness Assessment: HEALTH-RELATED QUALITY OF LIFE and UTILITY MEASURES. Final version [Internet]. Copenhagen: EUnetHTA, 2013.* 2015 (Adapted version).
23. European Network for Health Technology Assessment, *Endpoints used for Relative Effectiveness Assessment: SAFETY. Final version [Internet]. Copenhagen: EUnetHTA, 2013.* 2015 (Adapted version).
24. European Network for Health Technology Assessment, *Endpoints used for Relative Effectiveness Assessment: Surrogate Endpoints. Final version [Internet]. Copenhagen: EUnetHTA, 2013.* 2015 (Adapted version).
25. Wolowacz, S.E., et al., *Estimating health-state utility for economic models in clinical studies: an ISPOR good research practices task force report.* 2016. **19**(6): p. 704-719.
26. Berger, M.L., et al., *Good practices for real‐world data studies of treatment and/or comparative effectiveness: recommendations from the joint ISPOR‐ISPE Special Task Force on real‐world evidence in health care decision making.* 2017. **20**(8): p. 1003-1008.
27. Johnson, M.L., et al., *Good research practices for comparative effectiveness research: analytic methods to improve causal inference from nonrandomized studies of treatment effects using secondary data sources: the ISPOR Good Research Practices for Retrospective Database Analysis Task Force Report—Part III.* 2009. **12**(8): p. 1062-1073.
28. Cox, E., et al., *Good research practices for comparative effectiveness research: approaches to mitigate bias and confounding in the design of nonrandomized studies of treatment effects using secondary data sources: the International Society for Pharmacoeconomics and Outcomes Research Good Research Practices for Retrospective Database Analysis Task Force Report—Part II.* 2009. **12**(8): p. 1053-1061.
29. Berger, M.L., et al., *Good research practices for comparative effectiveness research: defining, reporting and interpreting nonrandomized studies of treatment effects using secondary data sources: the ISPOR Good Research Practices for Retrospective Database Analysis Task Force Report—Part I.* 2009. **12**(8): p. 1044-1052.
30. Ramsey, S., et al., *Good research practices for cost‐effectiveness analysis alongside clinical trials: the ISPOR RCT‐CEA Task Force report.* 2005. **8**(5): p. 521-533.
31. Mycka, J.M., et al., *Good research practices for measuring drug costs in cost effectiveness analyses: an industry perspective: the ISPOR drug cost task force report—Part V.* 2010. **13**(1): p. 25-27.
32. Hay, J.W., et al., *Good research practices for measuring drug costs in cost effectiveness analyses: issues and recommendations: the ISPOR Drug Cost Task Force Report—Part I.* 2010. **13**(1): p. 3-7.
33. Mansley, E.C., et al., *Good research practices for measuring drug costs in cost-effectiveness analyses: a managed care perspective: the ISPOR Drug Cost Task Force Report—part III.* 2010. **13**(1): p. 14-17.
34. Garrison Jr, L.P., et al., *Good Research Practices for Measuring Drug Costs in Cost‐Effectiveness Analyses: A Societal Perspective: The ISPOR Drug Cost Task Force Report—Part II.* 2010. **13**(1): p. 8-13.
35. Shi, L., et al., *Good Research Practices for Measuring Drug Costs in Cost‐Effectiveness Analyses: An International Perspective: The ISPOR Drug Cost Task Force Report—Part VI.* 2010. **13**(1): p. 28-33.
36. Mullins, C.D., et al., *Good research practices for measuring drug costs in cost‐effectiveness analyses: Medicare, Medicaid and other US government payers perspectives: the ISPOR Drug Cost Task Force report—Part IV.* 2010. **13**(1): p. 18-24.
37. Brazier, J., et al., *Identification, review, and use of health state utilities in cost-effectiveness models: an ISPOR good practices for outcomes research task force report.* 2019. **22**(3): p. 267-275.
38. Jansen, J.P., et al., *Indirect treatment comparison/network meta-analysis study questionnaire to assess relevance and credibility to inform health care decision making: an ISPOR-AMCP-NPC Good Practice Task Force report.* 2014. **17**(2): p. 157-173.
39. European Network for Health Technology Assessment, *Internal Validity of Non‐randomised Studies (NRS) on Interventions*. 2015, EunetHTA.
40. European Network for Health Technology Assessment, *Internal validity of randomised controlled trials*. 2015, EunetHTA.
41. Jansen, J.P., et al., *Interpreting indirect treatment comparisons and network meta-analysis for health-care decision making: report of the ISPOR Task Force on Indirect Treatment Comparisons Good Research Practices: part 1.* 2011. **14**(4): p. 417-428.
42. European Network for Health Technology Assessment, *Levels of Evidence: Applicability of evidence in the context of a relative effectiveness assessment of pharmaceuticals.* 2015.
43. Wailoo, A.J., et al., *Mapping to estimate health-state utility from non–preference-based outcome measures: an ISPOR good practices for outcomes research task force report.* 2017. **20**(1): p. 18-27.
44. European Network for Health Technology Assessment, *Meta-analysis of Diagnostic Test Accuracy Studies.* 2014.
45. European Network for Health Technology Assessment, *Methods for health economic evaluations - A guideline based on current practices in Europe.* 2015.
46. Briggs, A.H., et al., *Model parameter estimation and uncertainty: a report of the ISPOR-SMDM Modeling Good Research Practices Task Force-6.* 2012. **15**(6): p. 835-842.
47. Eddy, D.M., et al., *Model transparency and validation: a report of the ISPOR-SMDM Modeling Good Research Practices Task Force–7.* 2012. **32**(5): p. 733-743.
48. Caro, J.J., et al., *Modeling good research practices—overview: a report of the ISPOR-SMDM Modeling Good Research Practices Task Force–1.* 2012. **32**(5): p. 667-677.
49. Karnon, J., et al., *Modeling using discrete event simulation: a report of the ISPOR-SMDM Modeling Good Research Practices Task Force–4.* 2012. **32**(5): p. 701-711.
50. Wild, D., et al., *Multinational trials—recommendations on the translations required, approaches to using the same language in different countries, and the approaches to support pooling the data: the ISPOR patient-reported outcomes translation and linguistic validation good research practices task force report.* 2009. **12**(4): p. 430-440.
51. Thokala, P., et al., *Multiple criteria decision analysis for health care decision making—an introduction: report 1 of the ISPOR MCDA Emerging Good Practices Task Force.* 2016. **19**(1): p. 1-13.
52. Marsh, K., et al., *Multiple criteria decision analysis for health care decision making—emerging good practices: report 2 of the ISPOR MCDA Emerging Good Practices Task Force.* 2016. **19**(2): p. 125-137.
53. Benjamin, K., et al., *Patient-reported outcome and observer-reported outcome assessment in rare disease clinical trials: an ISPOR COA emerging good practices task force report.* 2017. **20**(7): p. 838-855.
54. Matza, L.S., et al., *Pediatric patient-reported outcome instruments for research to support medical product labeling: report of the ISPOR PRO good research practices for the assessment of children and adolescents task force.* 2013. **16**(4): p. 461-479.
55. Garrison Jr, L.P., et al., *Performance-based risk-sharing arrangements—good practices for design, implementation, and evaluation: report of the ISPOR good practices for performance-based risk-sharing arrangements task force.* 2013. **16**(5): p. 703-719.
56. European Network for Health Technology Assessment, *Personalised medicine and co-dependent technologies, with a special focus on issues of study design.* 2015.
57. European Network for Health Technology Assessment, *Practical considerations when critically assessing economic evaluations.* 2020.
58. Mauskopf, J.A., et al., *Principles of good practice for budget impact analysis: report of the ISPOR Task Force on good research practices—budget impact analysis.* 2007. **10**(5): p. 336-347.
59. Weinstein, M.C., et al., *Principles of good practice for decision analytic modeling in health-care evaluation: Report of the ISPOR task force on good research practices—Modeling studies.* 2003. **6**(1): p. 9-17.
60. Wild, D., et al., *Principles of good practice for the translation and cultural adaptation process for patient-reported outcomes (PRO) measures: report of the ISPOR task force for translation and cultural adaptation.* 2005. **8**(2): p. 94-104.
61. Eremenco, S., et al., *PRO data collection in clinical trials using mixed modes: report of the ISPOR PRO mixed modes good research practices task force.* 2014. **17**(5): p. 501-516.
62. European Network for Health Technology Assessment, *Process of information retrieval for systematic reviews and health technology assessments on clinical effectiveness.* 2019.
63. Berger, M.L., et al., *Prospective observational studies to assess comparative effectiveness: the ISPOR good research practices task force report.* 2012. **15**(2): p. 217-230.
64. Caro, J.J., et al., *Questionnaire to assess relevance and credibility of modeling studies for informing health care decision making: an ISPOR-AMCP-NPC Good Practice Task Force report.* 2014. **17**(2): p. 174-182.
65. Coons, S.J., et al., *Recommendations on evidence needed to support measurement equivalence between electronic and paper‐based patient‐reported outcome (PRO) measures: ISPOR ePRO Good Research Practices Task Force report.* 2009. **12**(4): p. 419-429.
66. Wang, S.V., et al., *Reporting to improve reproducibility and facilitate validity assessment for healthcare database studies V1. 0.* 2017. **20**(8): p. 1009-1022.
67. Marshall, D.A., et al., *Selecting a dynamic simulation modeling method for health care delivery research—Part 2: Report of the ISPOR Dynamic Simulation Modeling Emerging Good Practices Task Force.* 2015. **18**(2): p. 147-160.
68. Siebert, U., et al., *State-transition modeling: a report of the ISPOR-SMDM modeling good research practices task force–3.* 2012. **32**(5): p. 690-700.
69. Hauber, A.B., et al., *Statistical methods for the analysis of discrete choice experiments: a report of the ISPOR conjoint analysis good research practices task force.* 2016. **19**(4): p. 300-315.
70. McGhan, W.F., et al., *The ISPOR good practices for quality improvement of cost‐effectiveness research task force report.* 2009. **12**(8): p. 1086-1099.
71. European Network for Health Technology Assessment, *Therapeutic medical devices.* 2015.
72. Drummond, M., et al., *Transferability of economic evaluations across jurisdictions: ISPOR Good Research Practices Task Force report.* 2009. **12**(4): p. 409-418.
73. Rothman, M., et al., *Use of existing patient‐reported outcome (PRO) instruments and their modification: the ISPOR Good Research Practices for Evaluating and Documenting Content Validity for the Use of Existing Instruments and Their Modification PRO Task Force Report.* 2009. **12**(8): p. 1075-1083.
74. Drummond, M., et al., *Use of Pharmacoeconomics Information—Report of the ISPOR Task Force on Use of Pharmacoeconomic/Health Economic Information in Health‐Care Decision Making.* 2003. **6**(4): p. 407-416.
75. Garrison Jr, L.P., et al., *Using real‐world data for coverage and payment decisions: the ISPOR real‐world data task force report.* 2007. **10**(5): p. 326-335.
76. Zbrozek, A., et al., *Validation of electronic systems to collect patient-reported outcome (PRO) data—recommendations for clinical trial teams: report of the ISPOR ePRO Systems Validation Good Research Practices Task Force.* 2013. **16**(4): p. 480-489.
77. Fenwick, E., et al., *Value of information analysis for research decisions—an introduction: report 1 of the ISPOR Value of Information Analysis Emerging Good Practices Task Force.* 2020. **23**(2): p. 139-150.
78. Rothery, C., et al., *Value of information analytical methods: report 2 of the ISPOR value of information analysis emerging good practices task force.* 2020. **23**(3): p. 277-286.
79. Bridges, J.F., et al., *A roadmap for increasing the usefulness and impact of patient-preference studies in decision making in health: a good practices report of an ISPOR task force.* 2023. **26**(2): p. 153-162.
80. Mandrik, O.L., et al., *Critical appraisal of systematic reviews with costs and cost-effectiveness outcomes: an ISPOR good practices task force report.* 2021. **24**(4): p. 463-472.
81. Oortwijn, W., et al., *Designing and implementing deliberative processes for health technology assessment: a good practices report of a Joint HTAi/ISPOR task force.* 2022. **38**(1).
82. Orsini, L.S., et al., *Improving transparency to build trust in real-world secondary data studies for hypothesis testing—why, what, and how: recommendations and a road map from the real-world evidence transparency initiative.* 2020. **23**(9): p. 1128-1136.
83. Wang, S.V., et al., *HARmonized Protocol Template to Enhance Reproducibility of hypothesis evaluating real-world evidence studies on treatment effects: A good practices report of a joint ISPE/ISPOR task force.* 2022. **25**(10): p. 1663-1672.
84. Padula, W.V., et al., *Machine Learning Methods in Health Economics and Outcomes Research—The PALISADE Checklist: A Good Practices Report of an ISPOR Task Force.* 2022. **25**(7): p. 1063-1080.
85. Husereau, D., et al., *Consolidated Health Economic Evaluation Reporting Standards (CHEERS) 2022 explanation and elaboration: a report of the ISPOR CHEERS II good practices task force.* 2022. **25**(1): p. 10-31.

## Excluded articles

Ineligible articles (do not provide guidance for conduct of health economic evaluation or health technology assessment) = 21

1. Bertram, M.Y., et al., *Cost-effectiveness of population level and individual level interventions to combat non-communicable disease in Eastern Sub-Saharan Africa and South East Asia: a WHO-CHOICE analysis.* International journal of health policy management, 2021. **10**(11): p. 724.
2. Bertram, M.Y. and T.T.T. Edejer, *Introduction to the Special Issue on" The World Health Organization Choosing Interventions That Are Cost-Effective (WHO-CHOICE) Update".* International Journal of Health Policy Management, 2021. **10**(11): p. 670.
3. Ralaidovy, A.H., et al., *Priority setting in HIV, tuberculosis, and malaria–new cost-effectiveness results from WHO-CHOICE.* International Journal of Health Policy Management, 2021. **10**(11): p. 678.
4. Stenberg, K., et al., *Cost-effectiveness of interventions to improve maternal, newborn and child health outcomes: a WHO-CHOICE analysis for Eastern sub-Saharan Africa and South-East Asia.* International journal of health policy management, 2021. **10**(11): p. 706.
5. Institute for Clinical and Economic Review (ICER), *Guide to Understanding Health Technology Assessment (HTA).* 2018.
6. Frank C. Morris Jr and Alison Gabat, *ICER Analyses and Payer Use of Cost-effectiveness Results Based on the QALY and evLYG Are Consistent With ADA Protections for Individuals With Disabilities*. [No Year], Washington DC: Epstein Becker and Green P.C.
7. Seidner M, Emond SK, and Pearson SD, *Applying the Results of Comparative Effectiveness Research to Control Drug Costs: Policy Options for California*. 2023: Institute for Clinical and Economic Review (ICER).
8. Steven D. Pearson, et al., *Cornerstones of "fair" drug coverage: Appropriate cost-sharing and utilization management policies for pharmaceuticals*. 2020: Institute for Clinical and Economic Review (ICER).
9. Steven D. Pearson, et al., *Cornerstones of 'fair' drug coverage: appropriate cost sharing and utilization management policies for pharmaceuticals.* Journal of Comparative Effectiveness Research, 2021.
10. Caroline Pearson, Lindsey Schapiro, and Steven D Pearson, *The next generation of rare disease drug plicy: ensuring both innovation and affordability.* Journal of Comparative Effectiveness Research, 2022. **11**(14): p. 999-1010.
11. Caroline Pearson, Lindsey Schapiro, and Steven D Pearson, *The next generation of rare disease drug policy: Ensuring both innovation and affordability [report]*. 2022: Institute for Clinical and Economic Review (ICER).
12. Steven D Pearson, et al., *Assessment of Barriers to Fair Access*. 2021: Institute for Clinical and Economic Review (ICER).
13. Pearson SD, et al., *Assessment of Barriers to Fair Access*. 2023: Institute for Clinical and Economic Review.
14. Pearson SD, et al., *Assessment of Barriers to Fair Access: Supplemental Materials*. 2023: Institute for Clinical and Economic Review.
15. Kaltenboeck A, Mehlman A, and Pearson SD, *Strengthening the Accelerated Approval Pathway: An analysis of potential policy reforms and their impact on uncertainty, access, innovation and costs*. 2021: Institute for Clinical and Economic Review.
16. Kaltenboeck A, Mehlman A, and Pearson SD, *Potential policy reforms to strengthen the accelerated approval pathway.* Journal of Comparative Effectiveness Research, 2021. **10**(16): p. 1177-1186.
17. Rind DM, et al., *California Unsupported Price Increase Report: An Evaluation of Drug Price Increases During 2020 in California*. 2022: Institute for Clinical and Economic Review (ICER).
18. Rind DM, et al., *Unsupported Price Increase Report: Unsupported Price Increases Occurring in 2021*. 2022: Institute for Clinical and Economic Review.
19. Rind DM, et al., *Unsupported Price Increase Report: 2020 Assessment*. 2021: Institute for Clinical and Economic Review (ICER).
20. Rind DM, et al., *Unsupported Price Increase Report: Unsupported Price Increases Occurring in 2020*. 2021, Institute for Clinical and Economic Review (ICER).
21. Seidner M and Pearson SD, *Value Assessment and International Reference Pricing: Distinctive Strengths and Weaknesses as a Foundation for Medicare Drug Price Negotiation*. 2021: Institute for Clinical and Economic Review.

### Could not access = 1

1. Iran Ministry of Health, Iran Food and Drug Administration, National Committee for Selecting and Registering Medicine. Regulation of submitting applications for medicine with regard to development of economic evaluation. 2014

### Regional guidance = 1

1. Puig-Junoy J, Oliva-Moreno J, Trapero-Bertrán M, Abellán-Perpiñán M, Brosa-Riestra M, Servei Catalá de la Salut [CatSalut]. Guía y recomendaciones para la realización y presentación de evaluaciones económicas y análisis de impacto presupestario D. Sharma et al. demedicamientos en el ámbito del CatSalut. Barcelona: Generalitatde Catalunya, Departament de Salut, CatSalut; 2014.

### Not available in English and insufficient translation to interpret the data = 1

1. *Drug Economic Evaluation Guidelines [translated title]*. 2006, Seoul: Health Insurance Review Agency.
