# Supplementary material for: Incorporating healthcare access and equity in economic evaluations: a scoping review of guidelines
Source: Int J Technol Assess Health Care. 2024 Nov 18;40(1):e59. doi: 10.1017/S0266462324000618 (PMC11579673; doi:10.1017/S0266462324000618)
Supplement: Dawkins et al. supplementary material 3 — Dawkins et al. supplementary material [file S0266462324000618sup003.docx]

# Supplementary Appendix 3: Methods identified for analysis of healthcare access and equity

| **Method** | **Description** | **When to use it** | **Citations from this review** | **Known examples of use/ articles with additional information^1^** |
| --- | --- | --- | --- | --- |
| *Overall economic evaluation methods* | | | | |
| Cost-effectiveness analysis (CEA) | Analysis of the benefits of a healthcare intervention/ treatment relative to the costs. Provides estimate of the value of additional benefits of a new treatment. | This is the most common type of economic evaluation and is used to evaluate the cost-effectiveness of a given treatment or intervention. Some guidelines recommend robustly conducted cost-effectiveness analysis as being critical to achieving universal health coverage. | International guidelines: (1, 2) | Most common type of economic evaluation used in healthcare. |
| Generalised cost-effectiveness analysis (GCEA) | “GCEA is a form of CEA where a hypothetical reference case (“the null”) is used to identify the best package of interventions, regardless of previous, potentially inefficient, decisions.”(2) | To support development of universal healthcare coverage packages. | International guidance: (3) | Examples: (4-6) |
| Equity informative economic evaluation | Equity informative economic evaluation describes a collection of methods for economic evaluation which explicitly incorporate consideration of equity impacts alongside cost-effectiveness. Includes for example: Extended cost-effectiveness analysis (ECEA) and distributional cost-effectiveness analysis (DCEA) | When there are distributional equity impacts that should be considered quantitatively alongside cost-effectiveness. | Independent guidelines: (7) | Article describing equity informative cost-effectiveness analysis methods (8) |
| Distributional cost-effectiveness analysis (DCEA) | Analyses distribution of costs and effects within a population, estimates efficiency effects (cost-effectiveness) and equity effects. Identifies & analyses trade-offs between equity and efficiency e.g. using the equity-efficiency impact plane | Useful when comprehensive analysis of equity impacts is required and if there are potential trade-offs between equity and efficiency. | Independent guidelines: (7)   Methods guidance: (9) | Tutorial article: (10)  Book on DCEA methods: (11) |
| Aggregate DCEA | A less resource intensive version of DCEA. Can provide useful quantitative information to help policy makers consider how far new technologies are likely to reduce or increase health inequalities. | Useful when health inequality impacts need to be evaluated alongside population health, but available resources are not sufficient to undertake full DCEA. | Independent guidelines: (7) | Article evaluating 27 interventions in UK using aggregate DCEA framework (12) |
| Methods used within economic evaluations |  |  |  |  |
| Subgroup analysis | Analysing costs and effects that accrue to subgroups within the population. | Can be useful to demonstrate differential costs/effects for certain groups and highlight equity implications. Often used when there is suspected heterogeneity within the population. Generally advised that subgroups should be determined in advance of analysis based on known clinical or social characteristics that are expected to impact costs or effectiveness. | National guidelines: (13-38)   International guidelines: iDSI reference case  Independent guidelines: (1)  Methods guidance: (9, 39-48) | Example exploring gradient of hypertension by socio-economic status: (49)  Also many examples within published economic evaluations, e.g. (50) |
| Sensitivity analysis to explore effect of equity assumptions | Changing parameters used in primary analysis to reflect different equity stances and observing the effect on the results, e.g. scenario analysis. | If an equity approach is used in the primary analysis (e.g. as recommended by national guidance) but an alternative equity approach may be relevant to the setting or context. | National guidelines: (51)  Independent guidelines: (52) | Example: (53) |
| Absolute QALY shortfall weighting | “Absolute QALY shortfall is the future health, including quality and length of life, that is lost by people living with a condition, compared with the expected future health without the condition over the remaining lifetime of the patients.”(26) | When analysing interventions/treatments for severe conditions. | National guidelines: (26, 29) | Methods article including worked hypothetical example calculations: (54) |
| Equity/ distributional weighting/ adjustments | Costs/ outcomes are weighted according to equity relevant characteristics. One quantitative approach to determining weights is the "person trade-off approach" (55) | When an equity relevant characteristic is identified within the population and needs to be captured quantitatively within the analysis. | National guidelines: (20) (advises against use of this method)  International guidelines: (55)  Independent guidelines: (7)  Methods guidance: (56) | Quantitative weights can be used to give greater or lesser weight to subgroups of the population (57). |
| Demonstrate distributional effects | An extension to subgroup analysis where the population is split into groups representing a distribution according to an equity relevant characteristic and results are presented for each group in the distribution. | When an equity relevant characteristic is identified by which the population can be split, and differential costs/effects can be demonstrated across the distribution. | National guidelines: (27);  Independent guidelines: (7)   Methods guidance: (9) | Example estimating the impact of social investment policies on distribution of mortality & morbidity across age group categories & gender: (58) |
| Disaggregated societal perspective analysis | An analysis using a disaggregated societal perspective captures relevant non-health effects and costs that fall outside the health budget. | When there are relevant non health costs or effects. | International guidelines: (1) | Several examples of economic evaluations using this perspective, mainly from India: for example: (59) |
| The Health Improvement Distribution Index | Calculated as the disease prevalence in the subpopulation of interest divided by the disease prevalence in the overall population. | A simpler alternative to DCEA methods. | Independent guidelines: (7) | No published examples identified (non-systematic search). |
| Constrained optimisation | "A set of methods designed to identify efficiently and systematically the best solution (the optimal solution) to a problem characterized by a number of potential solutions in the presence of identified constraints," (Constrained optimization methods report 1). Allows budget/other constraints (e.g., equity) to be explicitly incorporated into the analysis. | When costs/ effectiveness is affected by identified constraints. These methods have been applied in problems of capacity management and location selection and may also be useful in guiding decision making where "physicians and patients face constraints such as proximity to treatment centers, health insurance benefit designs, and the limited availability of health resources." (Report 1) | Methods guidance: (60, 61) | Tutorial using Python: (62)  Examples: (63, 64) |
| Dynamic simulation modelling | Dynamic simulation models are a type of mathematical model used to represent operations or systems. They are different to other types of model widely used in healthcare evaluation (e.g. decision trees & Markov models) because they account for the dynamic nature of the system (changes over time) and the interactions between different system actors. Example types of dynamic simulation model: system dynamics, discrete event simulation and agent-based modelling. Machine leaning models may also complement these methods. | When additional constraints faced by patients and the health system need to be modelled to capture the impact on the patient pathway. E.g., capacity constraints, waiting times, utilisation and patient characteristics, and events can impact healthcare access. | Methods guidance: (65-67) | This article gives a good description of dynamic modelling approaches, what they can be used for and how to choose between them: (66) |
| Regression-based decomposition methods | Estimated regression coefficients are used to derive inequality weights of explanatory variables. | This method is useful to understand the determinants of inequality. | Methods guidance: (68) | These methods have been used widely in labour economics to investigate wage disparities by gender and race. For example: (69) More recently they have been used to examine racial disparities in access to healthcare. For example: (70) |
| Discrete event simulation (DES) | DES is a flexible modeling method characterized by the ability to  represent complex behaviour within, and interactions between individuals,  populations, and their environments. The term  “discrete” refers to the fact that DES models time in discrete intervals and that the events are mutually exclusive (discrete). | Useful when there are constrained resources and to account for queueing/ wait times; when 1) levels of access are altered; and 2) time to access impacts costs and outcomes | Methods guidance: (71, 72) | Example: (73) |
| *Methods used alongside economic evaluations* | | | | |
| Multi-Criteria Decision Analysis (MCDA) | A framework for incorporating multiple criteria within the decision-making process. Allows other social objectives (such as equity and access) be incorporated quantitatively into decision making alongside effectiveness/ cost-effectiveness. | When there are multiple dimensions of impact that need to be considered together within the decision-making process. Some concerns that MCDA can be too complicated to be practical. | Independent guidelines: (7, 74)  Methods guidance: (75) | Equity and other social implications were considered as one of 5 criteria in MCDA for universal coverage in Thailand. An MCDA tool also used in New Zealand to create a points system used for prioritising patients access to elective services. (75) |
| Report on equity according to PROGRESS-Plus | PROGRESS-Plus provides a framework for specifying indicators of social disadvantage: place of residence, race/ethnicity, occupation, gender, religion, education, social capital, socioeconomic status, plus age, disability and sexual orientation | A useful framework for describing equity impacts across the range of indicators of social disadvantage. | National guidelines: (31) | Example: (76) |
| Qualitative research to explore equity issues | Using qualitative research methods with key stakeholders to explore the impact of equity issues arising from a particular intervention. Examples from Philippine guidelines: participant observation, in-depth interviews, focus group discussions, and online ethnography | Useful to identify "factors and contexts, such as patient preferences, patient world view, social norms, socioeconomic status, gender roles, ethnicity, and religion that affect access to or quality of care received." (31) | National guidelines: (31, 77)  Independent guidelines: (74) | Examples: (78, 79) |
| Empirical measures to quantify equity impacts within a particular clinical condition or treatment | Empirical estimates to quantify equity impacts could be generated. These can be used to quantitatively describe equity impacts. | Can be useful when data/ resources are not sufficient to undertake DCEA but some quantification of equity would be useful to inform decision making. | Independent guidelines: (7) | Components of DCEA e.g. quantification of disease specific healthcare utilisation by equity variables such as age, sex socio economic status has been suggested as useful evidence for decision making even when full analysis of these equity distributions is not possible (7). |
| Conjoint analysis | Conjoint analysis is a decomposition  method, in which implicit values for an attribute  of an intervention are derived from some overall score for a  profile (conjointly) consisting of two or more attributes. Often involves a discrete choice experiment to elicit preferences. | Can be particularly useful for quantifying preferences for non-market goods or services or, as in the case of healthcare, where market  choices are severely constrained by regulatory and institutional factors Useful to assess patients' willingness to accept the therapeutic risks associated with more effective new treatments. Can help to explain adherence. | Methods guidance: (80) | Conjoint analysis has been used to measure preferences for a range of healthcare services e.g. (81-83)  For more examples see: (80) |
| ^1^References in this column were identified either within the documents included in the review or based on existing knowledge of the authors. They were not identified systematically and as such do not represent a complete list of examples or additional information. | | | | |

1. Wilkinson T, Sculpher MJ, Claxton K, Revill P, Briggs A, Cairns JA, et al. The international decision support initiative reference case for economic evaluation: an aid to thought. 2016;19(8):921-8.

2. Bertram MY, Lauer JA, Stenberg K, Edejer TTTJIJoHP, Management. Methods for the economic evaluation of health care interventions for priority setting in the health system: an update from WHO CHOICE. 2021;10(11):673.

3. Bertram MY, Lauer JA, Stenberg K, Ralaidovy AH, Edejer TT-TJIJoHP, Management. Progressive Realisation of Universal Health Coverage in Low-and Middle-Income Countries: Beyond the" Best Buys". 2021;10(11):697.

4. Bertram MY, Chisholm D, Watts R, Waqanivalu T, Prasad V, Varghese CJIjohp, et al. Cost-effectiveness of population level and individual level interventions to combat non-communicable disease in Eastern Sub-Saharan Africa and South East Asia: a WHO-CHOICE analysis. 2021;10(11):724.

5. Ralaidovy AH, Lauer JA, Pretorius C, Briët OJ, Patouillard EJIJoHP, Management. Priority setting in HIV, tuberculosis, and malaria–new cost-effectiveness results from WHO-CHOICE. 2021;10(11):678.

6. Stenberg K, Watts R, Bertram MY, Engesveen K, Maliqi B, Say L, et al. Cost-effectiveness of interventions to improve maternal, newborn and child health outcomes: a WHO-CHOICE analysis for Eastern sub-Saharan Africa and South-East Asia. 2021;10(11):706.

7. Agboola F, Whittington M, Pearson S. Advancing Health Technology Assessment Methods that Support Health Equity. 2023.

8. Cookson R, Mirelman AJ, Griffin S, Asaria M, Dawkins B, Norheim OF, et al. Using cost-effectiveness analysis to address health equity concerns. 2017;20(2):206-12.

9. Husereau D, Drummond M, Augustovski F, de Bekker-Grob E, Briggs AH, Carswell C, et al. Consolidated Health Economic Evaluation Reporting Standards (CHEERS) 2022 explanation and elaboration: a report of the ISPOR CHEERS II good practices task force. Value in health. 2022;25(1):10-31.

10. Asaria M, Griffin S, Cookson RJMDM. Distributional cost-effectiveness analysis: a tutorial. 2016;36(1):8-19.

11. Cookson R, Griffin S, Norheim OF, Culyer AJ. Distributional cost-effectiveness analysis: quantifying health equity impacts and trade-offs: Oxford University Press; 2020.

12. Love-Koh J, Cookson R, Gutacker N, Patton T, Griffin SJViH. Aggregate distributional cost-effectiveness analysis of health technologies. 2019;22(5):518-26.

13. Agenzia Italiana Del Farmaco. Guidelines for the compilation of the dossier in support of the request of refundability and price of a medicinal product [translated title]. 2020.

14. Daiga Behmane KL, Alar Irs, Nerimantas Steikunas. Baltic Guideline for Economic Evaluation of Pharmaceuticals (Parmacoeconomic Analysis). Baltic2002.

15. Secretariat of Science Technology and Strategic Inputs: Department of Science and Technology. Methodological Guidelines: Economic Evaluation Guideline (2nd Edition). Brazil: Ministry of Health; 2014.

16. Irina Cleemput MN, Stefaab Van De Sande, Nancy Thiry. Belgian Guidelines for Economic Evaluations and Budget Impact Analyses: Second Edition. Brussels: Belgian Healthcare Knowledge Centre (KCE); 2015.

17. Klimes J, Mlÿoch, T., Pásztor, B., Baloghová, K., Veselá, S., Bulejová. L., Tužil, J., Ornstová, E., Chadimová, K., Decker, B., Bartáková, J, Doležal, T.,. Recommended procedures for health economic evalutions in the Czech Republic: Czech Society for Pharmacoeconomics and Health technology evaluation [Translated title]. Czech Republic; 2020.

18. Danish Medicines Council. The Danish Medicines Council methods guide for assessing new pharmaceuticals. Denmark; 2021.

19. Pharmacoeconomic Unit: Central Administration for Pharmaceutical Affairs. Guidelines for Reporting Pharmacoeconomic Evaluations (Version: 01). Egypt: Egyptian Drug Authority, Ministry of Health; 2013.

20. Santé HAd. Methodological Guidance: Choices in Methods for Economic Evaluation. France; 2020.

21. Institute for Quality and Efficiency in Healthcare (IQWiG). General Methods: Draft version 7.0 [translated title]. Germany; 2022.

22. Ministry of Human Resources. Health professional guideline - For preparing and evaluating health economic analyses [translated title]. Hungary; 2021.

23. Health Information and Quality Authority. Guidelines for the Economic Evaluation of Health Technologies in Ireland. Ireland; 2020.

24. Centre for Outcomes Research and Economic Evaluation for Health (CORE2 HEALTH). Guideline for preparing cost-effectiveness evaluation to the central social insurance medical council. Japan: National Institute of Public Health; 2022.

25. Da Silva EA, Pinto CG, Sampaio C, Pereira J, Drummond M, Trindade RJL, Portugal: INFARMED. Guidelines for economic drug evaluation studies. Lisbon: National Authority of Medicines and Health Products [INFARMED],; 1998.

26. National Institute of Health and Care Excellence [NICE]. NICE health technology evaluations: the manual. London: NICE; 2022.

27. Inter-institutional Commission of the Basic Table of Inputs of the Health Sector. Guide for conducting economic evaluation studies to update the basics table and catalog of supplies of the health sector in Mexico [translated title]. Mexico: General Health Council; 2017.

28. Pharmaceutical Management Agency (New Zealand) (PHARMAC). Prescription for Pharmacoeconomic Analysis: Methods for cost-utility analysis (Version 2.2). New Zealand: PHARMAC; 2015.

29. The Norwegian Medicines Agency (NoMA). Guidelines for the submission of documentation for single technology assessment (STA) of pharmaceuticals. Norway; 2021.

30. CADTH Methods and guidelines. Guidelines for the Economic Evaluation of Health Technologies: Canada (4th Edition). Ottawa: CADTH; 2017.

31. Health Technology Assessment Unit DoH. Philippine HTA Methods Guide, First Edition. Republic of the Philippines: Department of Health; 2020.

32. Scottish Medicines Consortium (SMC). Guidance to submitting companies for completion of New Product Assessment Form (NPAF). Scotland: Healthcare Improvement Scotland; 2022.

33. Drug evaluation methods and process guide. Singapore: Agency for Care Effectiveness (ACE); 2019.

34. The Swedish Dental and Pharmaceutical Benefits Agency common advice. Sweden: The Swedish Dental and Pharmaceutical Benefits Agency; 2017.

35. Taiwan Society for Pharmacoeconomic Outcomes Research (TaSPOR). Guidelines of Methodological Standards for Pharmacoeconomic Evaluations in Taiwan (Version 1.0). Taiwan: TaSPOR; 2006.

36. Guideline for economic evaluations in healthcare. The Netherlands: Zorginstituut Nederland; 2016.

37. National Authority for Evaluation and Accreditation in Health (INEAS). Assessment of health technologies. Methodological choices for pharmaco-economic studies at INEAS [translated title]. Tunis: INEAS; 2021.

38. Pharmacy Practice and Development Division. Pharmacoeconomic Guidelines for Malaysia. Malaysia: Ministry of Health Malaysia; 2019.

39. European Network for Health Technology Assessment. Comparators & Comparisons. Criteria for the choice of the most appropriate comparator (s). Summary of current policies and best practice recommendations. Final version [Internet]. Copenhagen: EUnetHTA, 2013. 2015 (Adapted version).

40. Husereau D, Drummond M, Petrou S, Carswell C, Moher D, Greenberg D, et al. Consolidated health economic evaluation reporting standards (CHEERS)—explanation and elaboration: a report of the ISPOR health economic evaluation publication guidelines good reporting practices task force. Value in health. 2013;16(2):231-50.

41. European Network for Health Technology Assessment. Endpoints used for Relative Effectiveness Assessment: Clinical Endpoints. Final version [Internet]. Copenhagen: EUnetHTA, 2013. 2015 (Adapted version).

42. Cox E, Martin BC, Van Staa T, Garbe E, Siebert U, Johnson ML. Good research practices for comparative effectiveness research: approaches to mitigate bias and confounding in the design of nonrandomized studies of treatment effects using secondary data sources: the International Society for Pharmacoeconomics and Outcomes Research Good Research Practices for Retrospective Database Analysis Task Force Report—Part II. Value in Health. 2009;12(8):1053-61.

43. European Network for Health Technology Assessment. Methods for health economic evaluations - A guideline based on current practices in Europe. 2015.

44. Briggs AH, Weinstein MC, Fenwick EA, Karnon J, Sculpher MJ, Paltiel AD, et al. Model parameter estimation and uncertainty: a report of the ISPOR-SMDM Modeling Good Research Practices Task Force-6. Value in Health. 2012;15(6):835-42.

45. European Network for Health Technology Assessment. Practical considerations when critically assessing economic evaluations. 2020.

46. Mauskopf JA, Sullivan SD, Annemans L, Caro J, Mullins CD, Nuijten M, et al. Principles of good practice for budget impact analysis: report of the ISPOR Task Force on good research practices—budget impact analysis. Value in health. 2007;10(5):336-47.

47. Weinstein MC, O'Brien B, Hornberger J, Jackson J, Johannesson M, McCabe C, et al. Principles of good practice for decision analytic modeling in health-care evaluation: Report of the ISPOR task force on good research practices—Modeling studies. Value in health. 2003;6(1):9-17.

48. Drummond M, Brown R, Fendrick AM, Fullerton P, Neumann P, Taylor R, et al. Use of Pharmacoeconomics Information—Report of the ISPOR Task Force on Use of Pharmacoeconomic/Health Economic Information in Health‐Care Decision Making. Value in Health. 2003;6(4):407-16.

49. Tyroler HAJH. Socioeconomic status in the epidemiology and treatment of hypertension. 1989;13(5_supplement):I94.

50. Rheingans R, Anderson IV JD, Anderson B, Chakraborty P, Atherly D, Pindolia DJV. Estimated impact and cost-effectiveness of rotavirus vaccination in India: effects of geographic and economic disparities. 2014;32:A140-A50.

51. China Guidelines for Pharmacoeconomic Evaluations 2020 Edition. China: Sponsored by: Chinese Pharmaceutical Association, China Society for Pharmacoeconomics and Outcomes Research; 2020.

52. Institute for Clinical and Economic Review (ICER). 2020-2023 Value Assessment Framework: January 31, 2020 (Updated February 3, 2022). 2022.

53. McLeod M, Kvizhinadze G, Boyd M, Barendregt J, Sarfati D, Wilson N, et al. Colorectal cancer screening: how health gains and cost-effectiveness vary by ethnic group, the impact on health inequalities, and the optimal age range to screen. 2017;26(9):1391-400.

54. Versteegh MM, Ramos IC, Buyukkaramikli NC, Ansaripour A, Reckers-Droog VT, Brouwer WBJP. Severity-adjusted probability of being cost effective. 2019;37:1155-63.

55. Edejer TT-T, Baltussen R, Tan-Torres T, Adam T, Acharya A, Hutubessy R, et al. Making choices in health: WHO guide to cost-effectiveness analysis: World Health Organization; 2003.

56. Roberts M, Russell LB, Paltiel AD, Chambers M, McEwan P, Krahn M. Conceptualizing a model: a report of the ISPOR-SMDM modeling good research practices task force–2. Medical Decision Making. 2012;32(5):678-89.

57. Ward T, Mujica-Mota RE, Spencer AE, Medina-Lara AJP. Incorporating equity concerns in cost-effectiveness analyses: a systematic literature review. 2022:1-20.

58. Beckfield J, Morris KA, Bambra CJSjoph. How social policy contributes to the distribution of population health: the case of gender health equity. 2018;46(1):6-17.

59. Joshi BN, Shetty SS, Moray KV, Chaurasia H, Sachin OJBP, Childbirth. Cost-effectiveness and budget impact of adding tranexamic acid for management of post-partum hemorrhage in the Indian public health system. 2023;23(1):9.

60. Crown W, Buyukkaramikli N, Sir MY, Thokala P, Morton A, Marshall DA, et al. Application of constrained optimization methods in health services research: report 2 of the ISPOR optimization methods emerging good practices task force. Value in health. 2018;21(9):1019-28.

61. Crown W, Buyukkaramikli N, Thokala P, Morton A, Sir MY, Marshall DA, et al. Constrained optimization methods in health services research—an introduction: report 1 of the ISPOR optimization methods emerging good practices task force. Value in health. 2017;20(3):310-9.

62. Leung KB, Yousefi N, Chan TC, Bayoumi AMJMDM. Constrained Optimization for Decision Making in Health Care Using Python: A Tutorial. 2023:0272989X231188027.

63. Kapoor R, Standaert B, Nolan T, Pezalla E, Arnetorp S, Bergenheim K, et al. HSD27 Constrained Optimization Model to Estimate Best Booster Allocation Strategy to Minimize Hospital Bed-Days Under a Fixed Healthcare Budget. 2022;25(12):S278.

64. Buhat CAH, Lutero DS, Olave YH, Quindala KM, Recreo MGP, Talabis DAS, et al. Using constrained optimization for the allocation of COVID-19 vaccines in the Philippines. 2021;19:699-708.

65. Marshall DA, Burgos-Liz L, IJzerman MJ, Osgood ND, Padula WV, Higashi MK, et al. Applying dynamic simulation modeling methods in health care delivery research—the SIMULATE checklist: report of the ISPOR simulation modeling emerging good practices task force. Value in health. 2015;18(1):5-16.

66. Marshall DA, Burgos-Liz L, IJzerman MJ, Crown W, Padula WV, Wong PK, et al. Selecting a dynamic simulation modeling method for health care delivery research—Part 2: Report of the ISPOR Dynamic Simulation Modeling Emerging Good Practices Task Force. Value in health. 2015;18(2):147-60.

67. Padula WV, Kreif N, Vanness DJ, Adamson B, Rueda J-D, Felizzi F, et al. Machine Learning Methods in Health Economics and Outcomes Research—The PALISADE Checklist: A Good Practices Report of an ISPOR Task Force. Value in Health. 2022;25(7):1063-80.

68. Johnson ML, Crown W, Martin BC, Dormuth CR, Siebert UJViH. Good research practices for comparative effectiveness research: analytic methods to improve causal inference from nonrandomized studies of treatment effects using secondary data sources: the ISPOR Good Research Practices for Retrospective Database Analysis Task Force Report—Part III. 2009;12(8):1062-73.

69. Oaxaca RL, Ransom MRJJoe. On discrimination and the decomposition of wage differentials. 1994;61(1):5-21.

70. Zuvekas SH, Taliaferro GSJHa. Pathways to access: health insurance, the health care delivery system, and racial/ethnic disparities, 1996–1999. 2003;22(2):139-53.

71. Caro JJ, Briggs AH, Siebert U, Kuntz KM. Modeling good research practices—overview: a report of the ISPOR-SMDM Modeling Good Research Practices Task Force–1. Medical Decision Making. 2012;32(5):667-77.

72. Karnon J, Stahl J, Brennan A, Caro JJ, Mar J, Möller J. Modeling using discrete event simulation: a report of the ISPOR-SMDM Modeling Good Research Practices Task Force–4. Medical decision making. 2012;32(5):701-11.

73. England TJ, Harper PR, Crosby T, Gartner D, Arruda EF, Foley KG, et al. Examining the diagnostic pathway for lung cancer patients in Wales using discrete event simulation. 2021;10(3):1368.

74. Institute for Clinical and Economic Review (ICER). Modifications to the ICER value assessment framework for treatments for ultra-rare diseases: Final version, November 2017 (Updated January 31, 2020). 2020.

75. Thokala P, Devlin N, Marsh K, Baltussen R, Boysen M, Kalo Z, et al. Multiple criteria decision analysis for health care decision making—an introduction: report 1 of the ISPOR MCDA Emerging Good Practices Task Force. Value in health. 2016;19(1):1-13.

76. Attwood S, Van Sluijs E, Sutton SJIJoBN, Activity P. Exploring equity in primary-care-based physical activity interventions using PROGRESS-Plus: a systematic review and evidence synthesis. 2016;13:1-16.

77. National Institute of Health and Care Excellence [NICE]. Guide to the methods of technology appraisal 2013. London: NICE; 2013.

78. St. George SM, Harkness AR, Rodriguez-Diaz CE, Weinstein ER, Pavia V, Hamilton ABJIjoqm. Applying rapid qualitative analysis for health equity: lessons learned using “EARS” with Latino communities. 2023;22:16094069231164938.

79. Kaihlanen A-M, Virtanen L, Buchert U, Safarov N, Valkonen P, Hietapakka L, et al. Towards digital health equity-a qualitative study of the challenges experienced by vulnerable groups in using digital health services in the COVID-19 era. 2022;22(1):188.

80. Bridges JF, Hauber AB, Marshall D, Lloyd A, Prosser LA, Regier DA, et al. Conjoint analysis applications in health—a checklist: a report of the ISPOR Good Research Practices for Conjoint Analysis Task Force. 2011;14(4):403-13.

81. Weston A, FitzGerald PJP. Discrete choice experiment to derive willingness to pay for methyl aminolevulinate photodynamic therapy versus simple excision surgery in basal cell carcinoma. 2004;22:1195-208.

82. Coast J, Salisbury C, De Berker D, Noble A, Horrocks S, Peters T, et al. Preferences for aspects of a dermatology consultation. 2006;155(2):387-92.

83. King MT, Hall J, Lancsar E, Fiebig D, Hossain I, Louviere J, et al. Patient preferences for managing asthma: results from a discrete choice experiment. 2007;16(7):703-17.
